# Continuous Positive Linear Association between the Monocyte to High-Density Lipoprotein Cholesterol Ratio and Hypertension: A Cross-Sectional Study

**DOI:** 10.1155/2022/8501726

**Published:** 2022-12-08

**Authors:** Yaqing Zhou, Haijun Dan, Long Bai, Limei Jia, Baojin Lu, Guoqiang Gu, Wei Cui

**Affiliations:** ^1^Department of Physical Examination Center, The Second Hospital of Hebei Medical University, Shijiazhuang 050000, China; ^2^Department of Cardiology, The Second Hospital of Hebei Medical University and Institute of Cardiocerebrovascular Disease of Hebei Province, Shijiazhuang 050000, China

## Abstract

**Background:**

Hypertension poses a major threat to human health, and inflammation is associated with hypertension. The monocyte to high-density lipoprotein cholesterol ratio (MHR) represents a new inflammatory indicator. However, the relationship between the MHR and hypertension remains unclear. The present study investigated the association of MHR with hypertension.

**Method:**

For this cross-sectional study, we continuously collected data from the Physical Examination Centre of the Second Hospital of Hebei Medical University (*N* = 6632). The data included patients' demographic information and clinical information including blood pressure, blood biochemical measurements, and MHR. The relationship between the MHR and hypertension was examined using different methods in univariate and multivariate logistic analysis, smooth function analysis, the threshold saturation effect analysis and subgroup analysis.

**Results:**

The results showed that MHR was positively associated with hypertension without adjustment (odds ratio [OR] = 1.10, 95% confidence interval [CI]: 1.08–1.12, *P* < 0.001). The positive association still existed in minimally and fully adjusted models (OR = 1.08, 95% CI: 1.06–1.10, *P* < 0.001; OR = 1.07, 95% CI: 1.05–1.10, *P* < 0.001). Smooth function analysis of a generalized additive model revealed a continuous positive linear association between the MHR and hypertension throughout all MHR data (OR = 1.07, 95% CI: 1.05–1.10, *P* < 0.001). Subgroups analysis showed the homogeneity of the positive association among different subgroups.

**Conclusions:**

A continuous positive linear association was found between the MHR and hypertension in a health examination population.

## 1. Introduction

Hypertension is a significant risk factor for cardiovascular and cerebrovascular diseases, and as one of the most common chronic diseases worldwide, it represents a serious threat to public health. Predicting the occurrence of hypertension in the early stage, slowing the progress of hypertension, and preventing the onset of and improving the prognosis of cardiovascular and cerebrovascular diseases are crucial issues. It is well established that inflammation is associated with hypertension. Studies have shown that low-grade inflammation is a critical factor in elevated blood pressure [1]. Inflammation can lead to endothelial damage, increasing the adhesion of a variety of harmful substances and the recruitment of mononuclear phagocytes to the blood vessel walls, prompting the proliferation, motility, and differentiation of vascular smooth muscle cells, which can induce structural and functional modifications in the blood vessel walls, such as thickening, hardening, and elasticity reduction, to cause increased blood pressure [2]. Many studies have demonstrated close relationships between inflammatory markers and hypertension [3–5].

Most of these markers have proven to be a single action indicator with relatively poor stability, whereas the ratio of the monocyte count to the serum high-density lipoprotein cholesterol (HDL-C) level, referred to as the monocyte to HDL-C ratio and abbreviated MHR, provides a comprehensive indicator for evaluating dynamic changes in inflammation by combing the indicators of monocyte count and HDL-C level. Monocytes play an essential role in the inflammatory process related to hypertension. Under the action of chemokines, monocytes migrate, promote recruitment and activation of adhesion molecules, promote inflammation and oxidative stress, and participate in endothelial damage. In contrast, HDL-C resists inflammation and oxidative stress in addition to inhibiting the proliferation, differentiation, and activation of monocytes [6, 7]. The MHR can represent both the anti-inflammatory and antioxidative stress abilities along with the state and extent of inflammation, and this combined with its stability makes the MHR more advantageous than a single indicator. Moreover, measurements of monocyte counts and serum HDL-C levels are easy and inexpensive in clinical settings. Therefore, this study investigated the clinical value of the MHR as an index. Recent research has provided evidence that the MHR is associated with inflammatory conditions such as coronary artery disease [8] and stroke [9]. Notably, hypertension is also characterized by increased levels of metabolic and inflammatory markers, including the serum uric acid/HDL-C ratio [10] and triglyceride/HDL-C ratio [11]. Therefore, the monocyte/HDL-C ratio may also be associated with hypertension. However, the research on MHR has mainly focused on its use for the prognosis of cardiovascular and cerebrovascular diseases caused by hypertension [12–23]. The relationship between the MHR and hypertension has yet to characterized.

The present study aimed to characterize the relationship between the MHR and hypertension in a health examination population. First, we selected a new comprehensive inflammation indicator to explore the association with hypertension. Secondly, we selected a health examination population as the study population, as the association between the MHR and hypertension has never been explored in this particular population. Moreover, we strictly controlled confounding factors in multiple regression analyses using three adjusted models, used a generalized additive model to evaluate the relationship accurately and quantitatively, and performed subgroup analyses to further study the relationship in different subgroups. Altogether, we explored the relationship between the MHR and hypertension in a health examination from different perspectives.

## 2. Materials and Methods

### 2.1. Study Population

For this cross-sectional study, 8,428 participants who underwent physical examination at the Physical Examination Centre of the Second Hospital of Hebei Medical University were included in a consecutive manner from March 2017 to December 2017. A total of 1796 were excluded, including 87 who were younger than 18 years old, 555 for whom HDL-C levels were not recorded, 142 for whom the monocyte count was not available, 406 with diabetes, 256 with coronary heart disease, 271 with cerebrovascular disease, and 79 due to other serious illness. Finally, 6632 adults were retained as the research group for this study. The procedures performed were in accordance with the ethical standards of the Declaration of Helsinki and the responsible committees (Second Hospital of Hebei Medical University) for human experimentation. This study was approved by the Ethics Committee of the Second Hospital of Hebei Medical University (2021-P035).

The inclusion criteria included age ≥18 years and the availability of data from routine blood tests or blood lipid tests. The exclusion criteria included (1) age ≥75 years; (2) diagnosis of diabetes, coronary heart disease, cerebrovascular disease, and severe damage to liver and kidney function; (3) recent acute or chronic infectious disease or chronic autoimmune disease; (4) malignant tumours or blood system diseases; (5) current use of hormones and nonsteroidal anti-inflammatory drugs that could have an impact on the study; (6) taking an antilipemic medication that affects HDL-cholesterol levels; (7) a history of mental illness or use of psychotropic drugs; and (8) incomplete records from physical examination.

### 2.2. General Data Collection

For the study, physical examination data were collected for the population from the Second Hospital of Hebei Medical University, including general demographic data (name, age, sex, height, weight, systolic blood pressure [SBP], diastolic blood pressure [DBP], etc.), clinical information regarding medical history, and blood-related indicators (routine blood test results, liver and kidney function test results, blood lipid levels, etc.).

### 2.3. Diagnostic Criteria and Grouping Methods

According to the 2013 European Society of Cardiology (ESC)/European Society of Hypertension (ESH) guidelines, hypertension was defined as a SBP ≥140 mm Hg and/or DBP ≥90 mm Hg or lower blood pressure with the administration of antihypertensive medication [24].

For measurement of routine blood counts, blood lipid levels, and other blood indicators, 5 ml samples of venous blood were drawn from the elbow of participants in the morning after overnight fasting (7:00–9:00 am after more than 8 hours without food). First, the blood samples were centrifuged for 10 min at 1912 × *g* in a BY-600A centrifuge (Beijing Baiyang Medical Instrument Co., Ltd., Beijing, China), and serum was extracted. Blood routine tests were performed using a Beckman Coulter UniCel DxH 800 blood cell analyser (Beckman Coulter Inc., Brea, CA, USA) provided by the Clinical Laboratory of the Second Hospital of Hebei Medical University. Biochemical indicators such as liver and kidney function indexes and blood lipid levels were detected using a Beckman Coulter AU5800 automatic biochemical analyser (Beckman Coulter, Inc., Brea, CA, USA). The monocyte count and serum HDL-C concentration were used to obtain the MHR. The participants were then equally divided into three groups, which corresponded to MHR < 5.21, 5.21 ≤ MHR < 7.58, and MHR ≥ 7.58, respectively.

### 2.4. Statistical Methods

First the measurement data were tested for normality using the Shapiro–Wilk test. Data that followed a normal distribution were expressed as mean ± standard deviation (SD), and those that did not conform to the normal distribution were presented as median with interquartile range. Count data were expressed as percentage (%). One-way analysis of variance (ANOVA) was used to compare normally distributed clinical characteristics among the different MHR groups, while a nonparametric test was used to compare measurement data that did not follow a normal distribution. The chi-square test (*χ*^2^) and Fisher exact probability method were used for comparisons of classification variables. Univariate regression analysis was performed to analyse the relationships between general demographic data and hypertension. Then, multivariate logistic regression analysis was applied to explore the relationship between hypertension and the MHR considered as a continuous variable, a standardized continuous variable, a categorical variable, and continuous classification variables (sensitivity analysis) in an unadjusted model, minimally adjusted model (adjustment: sex and age), and fully adjusted model (adjustment: sex, age group, smoking history, drinking history, anemia, renal cyst, aspartate aminotransferase [AST] group, alanine aminotransferase [ALT] group, total bilirubin [TBIL] group, uric acid [UA] group, and estimated glomerular filtration rate [eGFR] group). Furthermore, the smoothing function of the generalized additive model was used to fit the potential nonlinear relationship between the MHR and hypertension, and the threshold saturation effect was applied to analyse whether a potential threshold or saturation effect existed between the MHR and hypertension. At the same time, after adjustment for confounding factors, subgroup analyses were performed to verify the relationship between the MHR and hypertension according to each subgroup stratification. Differences for which *P* < 0.05 were considered to be statistically significant. All statistical analyses were performed using the statistical software R.

## 3. Results

### 3.1. Demographic and Clinical Characteristics of Participants Stratified by MHR Quartiles

The study population consisted of 6632 adults with a mean age of 49.32 ± 10.96 years, including 3761 men (49.11 ± 10.87 years) and 2871 women (49.58 ± 11.07 years). The prevalence of hypertension among all participants was 31.76%.  Table 1 shows the demographic and clinical characteristics of the participants in different MHR groups. Significant differences were observed among the different MHR groups for most variables (*P* < 0.05). Compared with that in the *Q*1 group, the prevalence of hypertension in the *Q*3 group was significantly higher. In contrast, SBP and DBP in the *Q*3 group were considerably higher than those in the *Q*1 group (*P* < 0.001; Table 1).

### 3.2. Univariate Regression Analysis of the Relationship between MHR and Hypertension

On univariate analysis, the MHR was positively associated with hypertension, and the risk of hypertension increased by 10.00% with each increase in the MHR unit (odds ratio [OR] = 1.10, 95% confidence interval [CI]: 1.08–1.12, *P* < 0.001). Moreover, the risk of hypertension in *Q*3 group was 2.03 times higher than that in *Q*1 group (OR = 2.03, 95% CI: 1.78–2.31, *P* < 0.001; Table 2).

### 3.3. Multivariate Regression Analysis of the Relationship between MHR and Hypertension

As shown in Table 3, the results of multivariate regression analyses verified the relationship between the MHR and hypertension in the unadjusted model, the minimally adjusted model, and the fully adjusted model, separately, using four forms of MHR (continuous variable, standardized continuous variable, categorical variable, and continuous classification variables). The results showed that MHR was positively associated with hypertension in the crude model (OR = 1.10, 95% CI: 1.08–1.12, *P* < 0.001), and the positive association still was found after adjusting for confounding factors in the minimally adjusted model (OR = 1.08, 95% CI: 1.06–1.10, *P* < 0.001) and fully adjusted model (OR = 1.07, 95% CI: 1.05–1.10, *P* < 0.001). Moreover, each additional SD in MHR was associated with a 25% increase in the risk of hypertension in the fully adjusted model (OR = 1.25, 95% CI: 1.17–1.32, *P* < 0.001). When the MHR was converted to a categorical variable, the risk of hypertension in the *Q*3 group was 55% greater than that in the *Q*1 group (OR = 1.55, 95% CI: 1.33–1.81, *P* < 0.001). In all sensitivity analyses, the positive relationship still existed (*P* < 0.001).

### 3.4. Positive Linear Association between MHR and Hypertension

**Figure 1:**
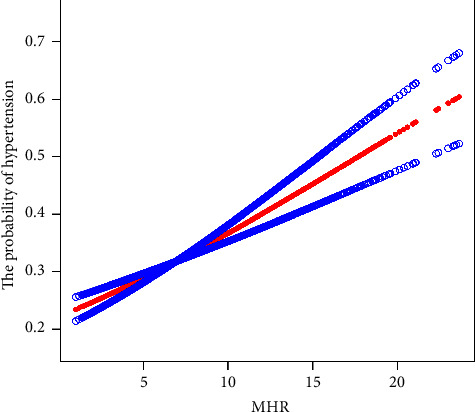
Positive linear association between MHR and hypertension. The red line represents the estimated probability of hypertension with increasing MHR. Blue bands represent 95% confidence intervals. Adjusted for sex, age group, alcohol drinking, smoking, anemia, renal cyst, eGFR group, UA group, AST group, ALT group, and TBIL group.

Because the MHR was a continuous variable, to further fit and analyse the potential nonlinear relationship between the MHR and hypertension, a smoothing function analysis of the generalized additive model was employed in this study. After adjusting the confounding factors, a continuous positive linear association was observed between the MHR and hypertension. Analysis of the threshold effect showed that the positive linear relationship between the MHR and hypertension continued through all MHR values among the study population (Table 4, Figure 1).

### 3.5. Subgroup Analyses of the Relationship between MHR and Hypertension

**Figure 2:**
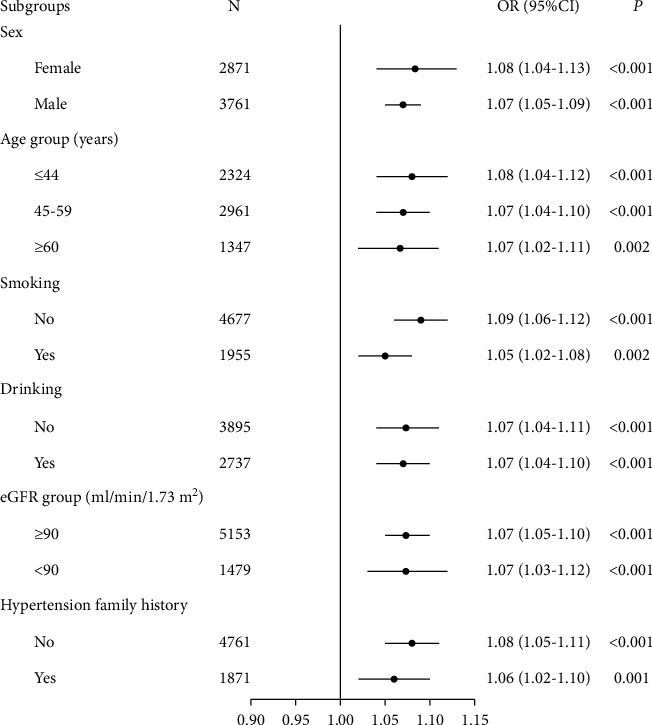
Subgroup analyses of the association of the MHR with hypertension after adjustment for confounding factors. Adjusted for sex, age group, alcohol drinking, smoking, anemia, renal cyst, eGFR group, UA group, AST group, ALT group, and TBIL group.

After an overall analysis, further subgroup analyses were performed to study the relationship between the MHR and hypertension. As shown in Figure 2, the relationship between the MHR and hypertension was analysed in different subgroups after adjustment of confounding factors. The results revealed homogeneity of the positive association was in further subgroups (*P* < 0.01).

## 4. Discussion

The present study comprehensively evaluated the association between the MHR and hypertension in a health examination population. The main results of the study are as follows: (1) The MHR was positively associated with hypertension with strict control for confounding factors. (2) A continuous positive linear association existed between the MHR and hypertension, and this linear relationship continued for all MHR data. (3) Subgroup analyses further showed the positive association was consistent in different subgroups.

Previous studies on MHR and hypertension have mainly focused on the MHR and hypertension target organ damage. Aydin et al. [13] confirmed that the MHR was positively associated with asymptomatic target organ damage in hypertension. Yayla et al. [25] found that the MHR in newly diagnosed and untreated hypertensive patients was significantly higher than that in healthy controls. Still, they did not further exclude confounding factors to analyse the actual relationship between the MHR and hypertension. Ji [26] investigated the MHR and asymptomatic target organ damage in patients with hypertension of varying severity, but performed only univariate analysis to analyse the relationship between the MHR and hypertension. Because the above studies focused on the MHR and target organ damage, the actual relationship between the MHR and hypertension was not studied. However, we explored the relationship between the MHR and hypertension from multiple directions and angles. The relationship between the MHR and hypertension was analysed in univariate and multivariate analyses. In multivariate analysis, the MHR was considered according to different data types. At the same time, the use of different multivariate regression analyses gradually eliminated confounding factors to analyse the relationship between the MHR and hypertension. Moreover, we used a generalized additive model to evaluate the relationship accurately and quantitatively, and subgroup analyses to further identify the relationship in different subgroups. Finally, a continuous positive linear association between the MHR and the risk of hypertension was observed and continued for all values of the MHR.

However, the underlying mechanism remains unclear. When an inflammatory response occurs in the body, LDL-C can enter into the vascular endothelium. The oxidized LDL-C can promote aggressive adhesion of monocytes to the vascular endothelium and can facilitate monocyte translation into macrophages, which can change into M1 and M2 phenotypes depending on inflammatory status. The M1 phenotype mainly leads to the occurrence of inflammation, which will maintain and aggravate the degree of hypertension. In comparison, the M2 phenotype can adjust the process of inflammation and cellular immune response, alleviating tissue damage and promoting organization repair [27–29]. Upon the occurrence of an inflammatory, M1 and M2 macrophages can switch functionally via regulation by the neuroimmune system, which causes a polarized state of macrophages that promotes permeation of cytokines into the central nervous system and stimulates the release of inflammatory factors locally. They pass the hypothalamus and its corresponding neural pathways and then connect nerve fibers directly to stimulate sympathetic nerves, which finally increases the blood pressure, leading to the development and progression of hypertension [30–32]. These findings show that LDL-C and monocytes/macrophages play an essential role in the development of hypertension. Different from the damaging effect of LDL-C on blood vessels, HDL-C has anti-inflammatory, antiapoptosis, and antiarteriosclerotic activities. Additional studies have demonstrated that HDL-C has anti-inflammatory and antioxidant effects and inhibits monocyte activation, proliferation, and differentiation, thus reducing the inflammatory response caused by monocytes [6, 7]. In addition, HDL-based markers play an important role in hypertension and related disorders. The serum uric acid/HDL ratio is associated with hypertension and other conditions that interact with hypertension, such as metabolic syndrome, type T2DM, and nonalcoholic fatty liver disease [10, 33–35]. Another marker, the triglyceride/HDL ratio, was found to be elevated in both hypertension and type T2DM [11, 36]. In the occurrence and development of hypertension, monocytes play the role of promoting inflammation and hypertension. In contrast, HDL-C acts to reduce inflammation and antagonize monocytes. Because the MHR combines monocytes and HDL-C, an increased MHR can represent an enhanced inflammatory response and a decrease in anti-inflammatory and antioxidant abilities, which may explain the mechanism by which a higher MHR is associated with increased blood pressure.

The present study offers the advantages of being one of the first studies on the MHR and hypertension at present. As a new inflammatory index MHR has a certain degree of novelty. Moreover, this study was an observational study, including inevitable potential confounding factors, and therefore, rigorous statistical methods were adopted to minimize the impact of confounding factors. In the analysis, the relationship between the MHR and hypertension was first analysed in an unadjusted model, a minimally adjusted model, and a fully adjusted model using four forms of MHR to represent the relationship between the MHR and hypertension more comprehensively. Secondly, this study used the smoothing function analysis of the generalized additive model to fit the potential nonlinear relationship of the MHR with hypertension to discover the true relationship between exposure and outcome. Finally, subgroup analyses further verified the relationship between the MHR and hypertension independent of a variety of clinical factors. The present study also has some limitations and deficiencies. First, this was a cross-sectional study, providing only weak evidence for the relationship between exposure and results and unable to determine the causal connection. Therefore, the causal relationship between the MHR and hypertension needs to be further verified in prospective studies. Second, some data were missing in the data collection process, including some medication use (antihypertensives and antihyperglycemics). The missing data could have led to a certain of results offset, affecting the relationship between the MHR and hypertension. While we excluded individuals taking antilipemic medication (statins and fibrates antilipemic) known to affect the HDL-cholesterol level, but some other medications, including antihypertensives and antihyperglycemics, might also have influenced the results. Moreover, this study did not include other inflammatory indicators, and the effects of the MHR and other inflammatory indicators on hypertension were not analysed. Data for other inflammatory indicators and complete medication use will be collected in our next study to further improve this research. At last, this study was conducted in a single-centre of a health examination population, which may have led to population selection bias and limiting the extension of the results to all groups. Therefore, further verification by multicentre, multiethnic, and large-scale clinical studies is still needed.

In conclusion, the MHR was positively associated with hypertension, and a continuous positive linear association was identified between the MHR and the risk of hypertension for all values of MHR.

## Figures and Tables

**Table 1:** Descriptive statistics of demographic and clinical characteristics in different MHR groups (*N* = 6632).

Characteristic	MHR			*P*
	*Q*1 (<5.21)	*Q*2 (≥5.21∼<7.58)	*Q*4 (≥7.58)	
*N*	2208	2213	2211	
Age (years)	49.56 ± 11.17	49.61 ± 10.94	48.78 ± 10.76	0.019
Monocyte count (×10^3^/mm^3^)	0.25 ± 0.06	0.33 ± 0.06	0.44 ± 0.11	<0.001
HDL-C (mg/dl)	63.79 ± 13.10	51.94 ± 9.50	43.23 ± 8.55	<0.001
SBP (mmHg)	123.37 ± 18.72	128.27 ± 18.78	130.89 ± 17.34	<0.001
DBP (mmHg)	75.49 ± 12.07	79.55 ± 12.34	82.11 ± 12.45	<0.001
Hb (g/L)	138.77 ± 15.38	145.82 ± 15.41	153.08 ± 13.97	<0.001
UA (*μ*mol/L)	273.53 ± 73.33	315.00 ± 81.45	349.71 ± 83.98	<0.001
eGFR (ml/min/1.73 m^2^)	99.83 ± 13.55	99.03 ± 13.32	98.87 ± 13.92	0.046
AST (U/L)	18.00 (15.00–22.00)	19.00 (15.00–23.00)	20.00 (16.00–25.00)	<0.001
ALT (U/L)	16.00 (11.00–22.00)	19.00 (13.00–26.00)	24.00 (17.00–35.00)	<0.001
TBIL (*μ*mol/L)	12.82 (10.13–16.27)	13.06 (10.23–16.77)	13.03 (10.20–16.72)	0.161
Sex				<0.001
Female	1510 (68.39%)	949 (42.88%)	412 (18.63%)	
Male	698 (31.61%)	1264 (57.12%)	1799 (81.37%)	
Age group (years)				0.051
≤44	763 (34.56%)	746 (33.71%)	815 (36.86%)	
45–59	981 (44.43%)	991 (44.78%)	989 (44.73%)	
≥60	464 (21.01%)	476 (21.51%)	407 (18.41%)	
Hypertension				<0.001
No	1680 (76.09%)	1496 (67.60%)	1350 (61.06%)	
Yes	528 (23.91%)	717 (32.40%)	861 (38.94%)	
Anemia				<0.001
No	2128 (96.38%)	2164 (97.79%)	2184 (98.78%)	
Yes	80 (3.62%)	49 (2.21%)	27 (1.22%)	
eGFR group (ml/min/1.73 m^2^)				0.135
≥90	1747 (79.12%)	1709 (77.23%)	1697 (76.75%)	
<90	461 (20.88%)	504 (22.77%)	514 (23.25%)	
Smoking				<0.001
No	1909 (86.46%)	1622 (73.29%)	1146 (51.83%)	
Yes	299 (13.54%)	591 (26.71%)	1065 (48.17%)	
Drinking				<0.001
No	1701 (77.04%)	1292 (58.38%)	902 (40.80%)	
Yes	507 (22.96%)	921 (41.62%)	1309 (59.20%)	
Renal cyst				<0.001
No	2120 (96.01%)	2125 (96.02%)	2073 (93.76%)	
Yes	88 (3.99%)	88 (3.98%)	138 (6.24%)	
AST group (U/L)				<0.001
<13	296 (13.41%)	252 (11.39%)	184 (8.32%)	
≥13∼<35	1787 (80.93%)	1810 (81.79%)	1843 (83.36%)	
≥35	82 (3.71%)	106 (4.79%)	155 (7.01%)	
NA	43 (1.95%)	45 (2.03%)	29 (1.31%)	
ALT group (U/L)				<0.001
<7	134 (6.07%)	73 (3.30%)	25 (1.13%)	
≥7∼<40	1933 (87.55%)	1888 (85.31%)	1753 (79.29%)	
≥40	98 (4.44%)	207 (9.35%)	404 (18.27%)	
NA	43 (1.95%)	45 (2.03%)	29 (1.31%)	
TBIL group (*μ*mol/L)				0.099
<17.10	1700 (76.99%)	1651 (74.60%)	1672 (75.62%)	
≥17.10	465 (21.06%)	517 (23.36%)	510 (23.07%)	
NA	43 (1.95%)	45 (2.03%)	29 (1.31%)	
UA group (*μ*mol/L)				<0.001
<357	2049 (92.80%)	1900 (85.86%)	1741 (78.74%)	
≥357	79 (3.58%)	232 (10.48%)	403 (18.23%)	
NA	80 (3.62%)	81 (3.66%)	67 (3.03%)	

**Table 2:** Univariate analysis of the associations of baseline characteristics with hypertension.

Covariate	Statistics	OR (95% CI)	*P*
Age (years)	49.32 ± 10.96	1.07 (1.06, 1.07)	<0.001
MHR	6.91 ± 3.10	1.10 (1.08, 1.12)	<0.001
Hb (g/L)	145.90 ± 16.04	1.02 (1.02, 1.03)	<0.001
UA (*μ*mol/L)	312.77 ± 85.57	1.00 (1.00, 1.01)	<0.001
eGFR (ml/min/1.73 m^2^)	99.24 ± 13.60	0.97 (0.96, 0.97)	<0.001
AST (U/L)	19.00 (15.00–24.00)	1.03 (1.02, 1.03)	<0.001
ALT (U/L)	19.00 (13.00–28.00)	1.02 (1.01, 1.02)	<0.001
TBIL (*μ*mol/L)	13.01 (10.18–16.57)	1.02 (1.01, 1.03)	<0.001
MHR group			
*Q*1 (<5.21)	2208 (33.29%)	Reference	
*Q*2 (≥5.21∼<7.58)	2213 (33.37%)	1.52 (1.34, 1.74)	<0.001
*Q*3 (≥7.58)	2211 (33.34%)	2.03 (1.78, 2.31)	<0.001
Sex			
Female	2871 (43.29%)	Reference	
Male	3761 (56.71%)	2.05 (1.84, 2.28)	<0.001
Age group (years)			
≤44	2324 (35.04%)	Reference	
45–59	2961 (44.65%)	2.73 (2.39, 3.12)	<0.001
≥60	1347 (20.31%)	5.95 (5.10, 6.94)	<0.001
Anemia			
No	6476 (97.65%)	Reference	
Yes	156 (2.35%)	0.46 (0.31, 0.70)	<0.001
eGFR group (ml/min/1.73 m^2^)			
≥90	5153 (77.70%)	Reference	
<90	1479 (22.30%)	2.07 (1.84, 2.34)	<0.001
Smoking			
No	4677 (70.52%)	Reference	
Yes	1955 (29.48%)	1.31 (1.18, 1.47)	<0.001
Drinking			
No	3895 (58.73%)	Reference	
Yes	2737 (41.27%)	1.70 (1.53, 1.88)	<0.001
Renal cyst			
No	6318 (95.27%)	Reference	
Yes	314 (4.73%)	2.24 (1.79, 2.81)	<0.001
AST group (U/L)			
<13	732 (11.04%)	Reference	
≥13∼<35	5440 (82.03%)	1.64 (1.37, 1.97)	<0.001
≥35	343 (5.17%)	2.70 (2.05, 3.56)	<0.001
NA	117 (1.76%)	1.18 (0.76, 1.86)	0.460
ALT group (U/L)			
<7	232 (3.50%)	Reference	
≥7∼<40	5574 (84.05%)	2.06 (1.47, 2.89)	<0.001
≥40	709 (10.69%)	3.13 (2.17, 4.52)	<0.001
NA	117 (1.76%)	1.56 (0.92, 2.66)	0.102
TBIL group (*μ*mol/L)			
<17.10	5023 (75.74%)	Reference	
≥17.10	1492 (22.50%)	1.21 (1.07, 1.36)	0.003
NA	117 (1.76%)	0.77 (0.51, 1.17)	0.223
UA group (*μ*mol/L)			
<357	5690 (85.80%)	Reference	
≥357	714 (10.77%)	2.13 (1.82, 2.50)	<0.001
NA	228 (3.44%)	0.67 (0.49, 0.92)	0.013

*Note.* CI, confidence interval; OR, odds ratio.

**Table 3:** Relationship between MHR and hypertension in different models.

Variable	Unadjusted model		Minimally adjusted model		Fully adjusted model	
	OR (95% CI)	*P*	OR (95% CI)	*P*	OR (95% CI)	*P*
MHR	1.10 (1.08, 1.12)	<0.001	1.08 (1.06, 1.10)	<0.001	1.07 (1.05, 1.10)	<0.001
MHR per-SD increase	1.35 (1.28, 1.42)	<0.001	1.26 (1.19, 1.34)	<0.001	1.25 (1.17, 1.32)	<0.001
MHR group						
*Q*1 (<5.21)	Reference		Reference		Reference	
*Q*2 (≥5.21∼<7.58)	1.52 (1.34, 1.74)	<0.001	1.35 (1.17, 1.55)	<0.001	1.28 (1.11, 1.48)	0.001
*Q*3 (≥7.58)	2.03 (1.78, 2.31)	<0.001	1.66 (1.43, 1.93)	<0.001	1.55 (1.33, 1.81)	<0.001
*P* for trend	<0.001		<0.001		<0.001	

*Note.* CI, confidence interval. OR, odds ratio. Effect: hypertension. Unadjusted model adjust for: none. Minimally adjusted model adjusted for: sex and age group. Fully adjusted model adjusted for: sex, age group, alcohol drinking, smoking, anemia, renal cyst, eGFR group, UA group, AST group, ALT group, and TBIL group.

**Table 4:** Analysis of threshold effect in the association between MHR and hypertension.

Exposure	MHR	
	OR (95%CI)	*P*
Model I		
One-line	1.07 (1.05, 1.10)	<0.001
Model II		
Turning point (T)	6.90	
Slope1: MHR < *T*	1.10 (1.04, 1.15)	<0.001
Slope2: MHR ≥ *T*	1.06 (1.03, 1.09)	<0.001
Slope2-Slope1	0.97 (0.91, 1.04)	0.379
A log likelihood ratio test	0.379	

OR, odds ratio, represented the effect for every 1 increase of MHR. Effect: hypertension. Cause: MHR. Adjusted for: sex, age group, alcohol drinking, smoking, anemia, renal cyst, eGFR group, UA group, AST group, ALT group, and TBIL group.

## Data Availability

The datasets generated during and/or analysed during the current study are available from the corresponding author upon reasonable request.

## Abbreviations

MHR: The monocyte to high-density lipoprotein cholesterol ratio
HDL: C high-density lipoprotein cholesterol
SBP: Systolic blood pressure
DBP: Diastolic blood pressure
AST: Aspartate aminotransferase
ALT: Alanine aminotransferase
TBIL: Total bilirubin
UA: Uric acid
eGFR: Estimated glomerular filtration rate
ANOVA: One-way analysis of variance
OR: Odds ratio
CI: Confidence interval
SD: Standard deviation.
